# A Functional Gene Array for Detection of Bacterial Virulence Elements

**DOI:** 10.1371/journal.pone.0002163

**Published:** 2008-05-14

**Authors:** Crystal Jaing, Shea Gardner, Kevin McLoughlin, Nisha Mulakken, Michelle Alegria-Hartman, Phillip Banda, Peter Williams, Pauline Gu, Mark Wagner, Chitra Manohar, Tom Slezak

**Affiliations:** 1 Chemistry, Materials, Earth and Life Sciences, Lawrence Livermore National Laboratory, Livermore, California, United States of America; 2 Computation, Lawrence Livermore National Laboratory, Livermore, California, United States of America; University of Wisconsin-Milwaukee, United States of America

## Abstract

Emerging known and unknown pathogens create profound threats to public health. Platforms for rapid detection and characterization of microbial agents are critically needed to prevent and respond to disease outbreaks. Available detection technologies cannot provide broad functional information about known or novel organisms. As a step toward developing such a system, we have produced and tested a series of high-density functional gene arrays to detect elements of virulence and antibiotic resistance mechanisms. Our first generation array targets genes from *Escherichia coli* strains K12 and CFT073, *Enterococcus faecalis* and *Staphylococcus aureus*. We determined optimal probe design parameters for gene family detection and discrimination. When tested with organisms at varying phylogenetic distances from the four target strains, the array detected orthologs for the majority of targeted gene families present in bacteria belonging to the same taxonomic family. In combination with whole-genome amplification, the array detects femtogram concentrations of purified DNA, either spiked in to an aerosol sample background, or in combinations from one or more of the four target organisms. This is the first report of a high density NimbleGen microarray system targeting microbial antibiotic resistance and virulence mechanisms. By targeting virulence gene families as well as genes unique to specific biothreat agents, these arrays will provide important data about the pathogenic potential and drug resistance profiles of unknown organisms in environmental samples.

## Introduction

Rapid detection and characterization of bacterial and viral pathogens has become a vital component of our national biodefense strategy. Various detection technologies based on nucleic acid signatures have emerged in the past few years, including TaqMan and Luminex bead based systems. While these technologies are able to rapidly identify selected pathogens at the species or strain level, they do not have the capability to provide broad functional information about known or novel organisms. Characterization of emerging, engineered, or unknown pathogens requires a platform that can assess the virulence and antibiotic resistance mechanisms present in these organisms. One recent approach that has been used successfully to measure other types of microbial capabilities is known as a functional gene array (FGA).

A functional gene array is a DNA microarray containing probes targeting sequences unique to genes within families of interest. Family-specific probes are designed to match regions that are conserved among genes in the family, in order to increase the chance of detecting previously unidentified homologs. Small-scale FGAs have been used successfully to measure the presence and activity of key enzymes in environmental samples [1]. The largest functional gene array described to date contains 1,662 50-mer oligonucleotide probes for 2,402 genes involved in biodegradation and metal resistance [2], and was recently upgraded to include over 24,000 probes [3]. More recently FGAs have been applied in the area of molecular and clinical diagnostics for pathogens [4].

The FGAs developed to date have focused on specific sets of gene functions, thereby limiting their use to narrowly defined applications. Because of the broad diversity of pathogens and the large number of gene families involved, constructing a functional gene array to detect genes associated with virulence and antibiotic resistance is a much greater challenge. We define virulence-related genes as those whose products affect the ability of a pathogen to infect or survive in the host, are required for expression of other virulence factors, or cause the host direct harm (such as toxins). A high-density oligonucleotide microarray is the only platform available at present that supports simultaneous interrogation of such a wide variety of genes. The approach of using presence or absence of virulence genes as a forensic classifier has been demonstrated in a recent study that used PCR to differentiate several *E. coli* strains [5]. However, the small number of genes that can be measured per assay remains a limitation of PCR-based techniques.

Our laboratory has a NimbleGen array synthesizer that is capable of making arrays with up to 388,000 probes per array, with variable probe lengths ranging from 23 to 85 nucleotides (nt) [6]. This has made it possible for our group to prototype a series of arrays for experimentation to find the optimal probe design parameters for detecting signatures of functional gene families.

The other major challenge in constructing functional arrays for detecting virulence genes is the exhaustive computation required to design sensitive and specific probes for hundreds of thousands of gene target sequences. Millions of sequence comparisons are required to find the most conserved regions within gene families and subfamilies, to ensure that probes are selected to span diverse gene sequences that encode similar functions. Thermodynamic binding energy predictions, conservation and uniqueness scores must be computed for millions of candidate probes, in order to select an optimal combination of probes for each target gene family, balancing sensitivity, specificity, and breadth of coverage. The computation of each of these factors is CPU-intensive, requiring that we develop highly efficient algorithms and implement them using high performance computers (HPC) at Lawrence Livermore National Laboratory (LLNL). Our access to HPC facilities has played a crucial role in making high-quality probe design and selection feasible at this scale.

In this report, we describe the process used to design our first generation functional array for highly sensitive detection of virulence and antibiotic resistance gene families. We discuss the probe design algorithms, including virulence gene sequence selection, and our protocols for sample preparation, amplification, labeling, hybridization, and data analysis. We present the results from experiments designed to assess whether the array can detect virulence gene orthologs from organisms without perfect match probes on the array, using both targeted mismatch probes and hybridizations to DNA from other organisms. Also, we report the results from limit of detection studies, using known amounts of bacterial DNA spiked into aerosol samples to measure the minimal concentration required for detection of virulence elements against a complex background.

## Methods

### Virulence gene sequence selection

Gene target sequences were selected from the genomes of the four bacterial strains shown in Table 1. These strains were selected because they were commercially available, have genome sequences published in GenBank [7], and required no more than a biosafety level 2 laboratory for sample processing. In addition, each has representatives of a wide variety of virulence-related gene families. Because our study focused on designing robust, sensitive probes for gene families, our target sequence set includes virulence gene orthologs found in strains such as *E. coli* K12 that are avirulent to humans.

**Table 1:** Bacterial Strains Used for Probe Selection

Species	Strain	GenBank Accession
*Escherichia coli*	CFT073	AE014075.1
*Escherichia coli*	K-12 MG1655	U00096.2
*Enterococcus faecalis*	V583	AE016830.1
*Staphylococcus aureus* subsp. *aureus*	Mu50	NC_002758.2

We selected target sequences from the four genomes by searching for virulence-related proteins using 712 sets of profile hidden Markov models (HMMs). HMM sets were designed or selected by Swan *et al*. [8] to recognize a collection of several hundred virulence-associated protein families identified from the literature and public databases. In most cases the HMM sets consisted of single profile HMMs; however, to distinguish between related families that share common protein domains, it was sometimes necessary to combine HMMs for a sequence of protein domains. For these HMM sets, the score assigned to a candidate sequence was the sum of the individual HMM bit scores, and a sequence was only considered a match if all the component domains were present in the correct order. In order to find optimal HMM sets, we designed multiple HMM sets for certain virulence protein families.

We found matches for 299 of the HMM sets in the four genomes, corresponding to 160 protein families. Some families were represented by multiple paralogs in a given genome, and others by none; in addition, HMM sets within a family sometimes matched distinct but overlapping target sequences, yielding a total of 1,245 matching sequences. The search was performed using the “estwisedb” algorithm of the Wise 2.0 software [9] on the Thunder supercomputer at LLNL (http://www.llnl.gov/pao/news/news_releases/2007/NR-07-04-05.html). For all the predicted gene sequences, existing gene annotations were downloaded from GenBank and correlated with the coordinates of the HMM matches. HMM hits for the same gene family in multiple strains of the same organism had very similar if not identical sequences for all the cases examined.

### Probe design for virulence gene target sequences

**Figure 1:**
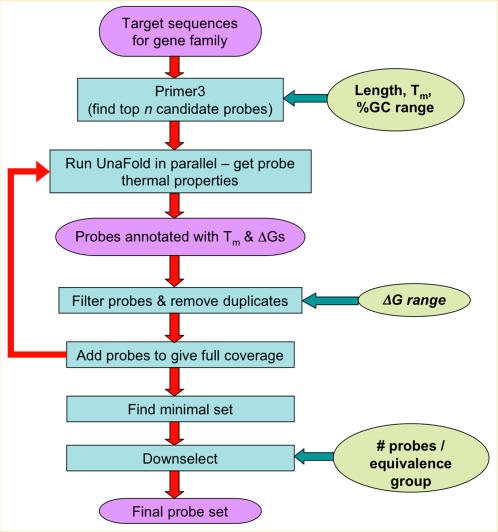
Virulence array probe design process. Candidate probes were generated using Primer3 and Unafold based on *T_m_*, GC content, salt concentration, and minimum free energies of probes. Probes were filtered based on best free energy and duplicate probe sequences were removed. When necessary, additional candidate probes were generated using more relaxed parameters to ensure full coverage. The final set of probes was then downselected to produce a maximum of 30 probes per equivalence group, each capable of detecting multiple target sequences in a given family.

After selecting and extracting target gene sequences, we designed probes as diagrammed in Figure 1. In summary, we selected probes for a given gene family using a greedy algorithm favoring the most conserved regions of sequences within that family, while ensuring that each target sequence had a minimum number of probes that were complementary to it. More details follow below. Using the most conserved regions enabled coverage of more sequences with fewer probes, and thus detection of more potential families on a single array, than simply tiling probes across each target sequence. We included additional probes for divergent sequences not captured by the conserved probes so that all known orthologs within the 4 genomes could be detected.

In the first step of probe design, we generated candidate probes from all four organisms for the target gene sequences in a given family using MIT's Primer3 software [10]. We selected possible probe candidates based on the rough predictions of the melting temperature *T_m_* derived by Primer3 from the length, percent GC content and salt concentration. The parameters used for Primer3 are shown in Table 2.

**Table 2:** Input parameters for Primer3 probe generation

Primer3 parameter	Value
PRIMER_TASK	pick_hyb_probe_only
PRIMER_PICK_ANYWAY	1
PRIMER_INTERNAL_OLIGO_OPT_SIZE	60
PRIMER_INTERNAL_OLIGO_MIN_SIZE	50
PRIMER_INTERNAL_OLIGO_MAX_SIZE	66
PRIMER_INTERNAL_OLIGO_OPT_TM	90
PRIMER_INTERNAL_OLIGO_MIN_TM	80
PRIMER_INTERNAL_OLIGO_MAX_TM	150
PRIMER_INTERNAL_OLIGO_MIN_GC	25
PRIMER_INTERNAL_OLIGO_MAX_GC	75
PRIMER_EXPLAIN_FLAG	0
PRIMER_INTERNAL_OLIGO_SALT_CONC	450
PRIMER_INTERNAL_OLIGO_DNA_CONC	100
PRIMER_INTERNAL_OLIGO_MAX_POLY_X	4
Other parameters	defaults

We next used a modified version of Unafold [11] to make more accurate predictions of *T_m_* and the minimum free energies of probe-target hybridization (Δ*G_complement_*), probe–probe hybridization (Δ*G_homodimer_*), and probe–self hybridization (Δ*G_hairpin_*). While Unafold is a highly accurate program for *ΔG* and *T_m_* prediction, it was too slow given our need to calculate *T_m_*'s and *ΔG*'s for millions of candidate probes. We created an accelerated version of Unafold that ran more than ten times faster by using more efficient data structures and caching thermodynamic parameter tables in memory rather than reloading them for each probe. We then used the predicted *ΔG*'s to compute an aggregate “Δ*G_adjusted_*” (described below) for each candidate probe. Candidate probes with unsuitable *ΔG*'s or T_m_'s were excluded, unless fewer than 15 candidate probes per target sequence passed all the thermodynamic criteria described above; in this case, candidate probes that failed the filters were included to ensure at least 15 candidates per target.

After this initial screening step, we used a custom Perl program to cluster target sequences within each gene family into overlapping “equivalence groups”. Equivalence groups are sets of targets sharing one or more common sequence regions from which probes may be drawn. We implemented a greedy algorithm to select probes from the minimal set of equivalence groups that covered all target sequences in a gene family. The algorithm searches all equivalence groups for a family, finding in each iteration the group containing the largest number of target sequences not already represented by a sufficient number of probes. Probes are selected from the shared sequences in this equivalence group, and the search is repeated until all targets are covered by a minimum number of 15 probes. By prioritizing equivalence groups in this way, the selection process favors probes for regions that are more highly conserved in a gene family.

When an equivalence group contained more than 30 candidate probes, we used a custom Python downselection program to choose an optimal subset. The downselection program uses an iterative ranking algorithm favoring probes having lower (more negative) Δ*G_adjusted_* and greater dispersal across the target gene sequence.

This process of generating candidate probes, clustering targets into equivalence groups, choosing a minimal set of equivalence groups, and downselecting probes within equivalence groups, was repeated for the target sequences from each gene family.

In addition, we included 2,000 positive control probes from the four genomes. These were designed to be distributed widely across each genome, and to range in length between 50–66 nt, in GC content between 40–60%, and in *T_m_* between 71–91°C.

### Probe design parameter optimization

Detection of pathogens or gene families represented in a genomic DNA sample requires different probe design criteria than those used for gene expression, ChIP-chip or resequencing purposes. Each target type requires an appropriate balance between probe sensitivity and specificity. Ideally, all probes should be sensitive enough to detect DNA at single-copy concentrations. However, probes intended to distinguish organisms at the species or strain level must be designed to avoid cross-hybridization; while probes used to detect the presence of gene families should allow some degree of mismatch between the probe and target sequence, congruent with the range of sequence variation among orthologs within the family.

In order to determine the effect of design parameters on probe sensitivity and specificity, we constructed a prototype array containing several hundred probes for each member of ten gene families having representatives in all four target species. For each target gene, probes were selected spanning a wide range of design parameters. Probe lengths ranged from 30 to 66 nt, GC content from 40% to 60%, predicted *T_m_* from 66°C to 91°C, Δ*G_complement_* from −30 to −92 kcal/mol, Δ*G_homodimer_* from −1.5 to −12 kcal/mol, and Δ*G_hairpin_* from +1.8 to −6 kcal/mol. Prototype arrays were synthesized and hybridized to 4 μg of pure genomic DNA from one of the four species, as described below.

**Figure 2:**
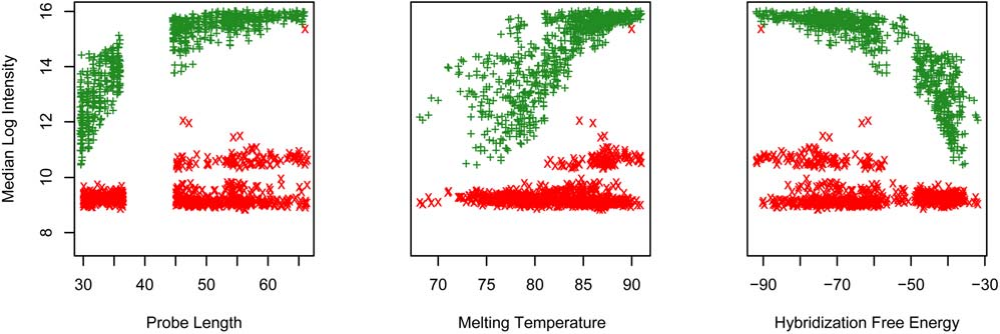
Log_2_ intensity vs probe length, predicted melting temperature *T_m_*, and predicted complement ΔG for selected probes in an array hybridized with *E. coli* CFT073 genomic DNA. Probes specific for *E. coli* sequences are plotted in green; probes specific for *E. faecalis* are in red.

Figure 2 shows log signal intensities for probes targeting a typical gene family, in which DNA complementary to one set of probes (those for *E. coli* CFT073) was present in the hybridization mix, while DNA for another set of probes (for *E. faecalis)* was absent; thus the signal seen for *E. faecalis* probes is entirely due to non-specific hybridization and other sources of background noise. We found that probes with lengths above 50 nt gave significantly stronger signals, with better differentiation from background, than lengths in the 30 to 45 nt range. The predicted melting temperature and Δ*G_complement_* are strongly correlated with probe length, but not entirely determined by it. We performed linear regression fits to the log intensity against each of the probe design parameters, and multiple regressions against several combinations of parameters. Of the individual probe parameters we examined, the best predictor of intensity (i.e., the one with the smallest residual variance) was Δ*G_complement_*; the best multivariate predictor was a combination of Δ*G_complement_*, Δ*G_homodimer_*, and Δ*G_hairpin_*.

**Figure 3:**
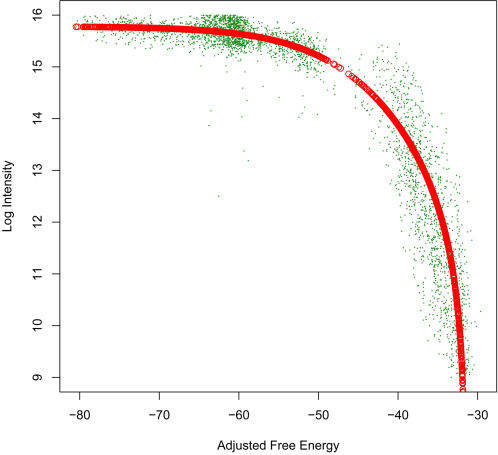
Langmuir isotherm fit of adjusted ΔG vs median log intensity of one array. A linear combination of the three free energies (Δ*G_complement_*, Δ*G_homodimer_* and Δ*G_hairpin_*) which we term “Δ*G_adjusted_*” was the best predictor of hybridization intensity for probes complementary to the target DNA.

We observed that the relationship of log intensity to thermodynamic parameters such as Δ*G_complement_* is nonlinear, and shows evidence of chemical saturation for the most sensitive probes. In order to incorporate saturation into our probe response model and find the combination of thermodynamic parameters that was the best predictor of probe sensitivity, we fit our data to a Langmuir isotherm curve [12] (Figure 3), parameterized by the Δ*G_complement_*, Δ*G_homodimer_*, and Δ*G_hairpin_* as follows:
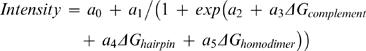
We performed a nonlinear least squares fit to data from eight microarrays, each hybridized to 1–5 μg of DNA from one of the four target species, to fit values for the parameters *a_0_* through *a_5_.* We determined that a linear combination of the three free energies which we term “Δ*G_adjusted_*” was the best predictor of hybridization intensity for probes complementary to the target DNA. The Δ*G_adjusted_* is defined as:
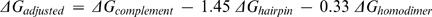



In subsequent array designs, we screened candidate probes to include only those with predicted *T_m_*≥80°C, Δ*G_homodimer_*>−12 kcal/mol, Δ*G_hairpin_*>−6 kcal/mol, and Δ*G_adjusted_*≤−55 kcal/mol.

### Mismatch probe permutation methods

We generated mismatch (MM) probes, derived from a selection of perfect match (PM) target probe sequences, in order to test the ability of probes designed against gene family members from one organism to detect orthologs with non-identical sequences from other organisms. Previous experiments suggested that hybridization to MM probes depends strongly not only on the number of mismatched bases, but also on their location and distribution across the length of the probe (data not shown). MM probes were generated using five different strategies, incorporating single, adjacent, random, interval, and shifting PM region mismatches. Single and adjacent MM probes were generated by sliding a window of size *k* (with *k* taking values 1, 2, 3, 6, 10, 15 and 20) across the PM sequence, and creating *k* mismatches at the location of the window. We generated random MM probes by selecting *k* random positions in the PM probe, with *k* = 1, 2, 3, 6, 10, 15 or 20, and creating single MM at each position. In interval MM probes, mismatches were placed at regular intervals of size *k*, starting with a MM at the first base at the 5′ end of the probe. MM probes with shifting PM regions were created for region lengths *n* between 15 and 30, and offset values *s* ranging from 2 to 29. For each combination of length and offset, a probe was generated by preserving a PM region of size *n*, starting at base position *s*, and creating a MM at every third base on either side of the PM region.

### Random control probe design

We generated 3,000 negative control probes, consisting of random sequences designed to have the same distribution of length and GC content as the target probes. BLAST searches of the random probes against the GenBank nt database showed that none had any perfect match alignments of length greater than 21 nt to any known sequence, so that none would be expected to hybridize to the organisms we tested on the arrays. These were used to determine background noise levels due to non-specific hybridization of target DNA and to fluorescence of the chip substrate.

### Microarray synthesis

DNA microarrays were prepared on glass microscope slides according to a photolabile deprotection strategy that has been previously described [6]. Arrays were generated at the LLNL Microarray Center. Reagents and supplies for the microarray syntheses were purchased from Roche NimbleGen (Madison, WI). Between 3 and 5 replicate features were generated for each probe and randomly assigned to locations on the array. 388,000 features were produced per array using a checker-board pattern leaving every other spot vacant. The final deprotection and quality control of the arrays were carried out as described [13]. Each array contained approximately 3,000 24-mer *Arabidopsis* calmodulin protein kinase 6 (CPK6) fiducial spots. The slides were hybridized with complementary CPK6-Cy3 (Integrated DNA Technologies, Coralville, IA) and scanned to assure the quality of each array before hybridizing to DNA targets.

### Sample preparation and microarray hybridization

*E. coli* K12 MG1655, *E. coli* CFT073, *E. faecalis* V583 and *S. aureus* Mu50 were purchased from ATCC. The bacterial culture pellets were grown according to the instructions from ATCC and genomic DNA was extracted using the Epicentre DNA extraction kit according to the manufacturer's protocols. The DNA was quantified using a NanoDrop spectrophotometer (Wilmington, DE). DNA samples were sonicated to fragment the DNA to a size range of 500–2000 bp, and then labeled using nick translation with Cy3-labeled random nonamer primers (TriLink Biotechnologies, San Diego, CA) and Klenow DNA polymerase at 37°C for 3 hr. The labeled DNA was precipitated in isopropanol, and the pellet was washed, dried, reconstituted and quantified. For each hybridization, 4 μg of labeled DNA was mixed with Cy3-labeled CPK6 oligomers, NimbleGen hybridization components and hybridization buffer according to the manufacturer's protocols. The arrays were hybridized with labeled DNA on a MAUI hybridization station (BioMicro Systems, Salt Lake City, UT) at 42°C for 16 hr. Arrays were washed with NimbleGen wash buffers I, II and III according to vendor protocols and scanned using an Axon GenePix 4000B scanner at 5 μm resolution.

For limit of detection experiments, aerosol filters were kindly supplied by the BioWatch program and DNA was extracted using the MoBio UltraClean Soil DNA kit (MoBio Laboratories, Carlsbad, CA), as described in [14]. DNA from one filter was used as a common background, to which varying quantities of fragmented *S. aureus* DNA were added. *S. aureus* DNA was quantified using a Quant-iT PicoGreen ds DNA kit (Invitrogen, Carlsbad, CA), serially diluted, and then spiked into 10 ng of aerosol sample DNA in quantities of 0.31 fg, 3.1 fg, 31 fg, 310 fg or 3.1 pg. We performed whole genome amplification of the combined samples (aerosol samples with spiked-in *S. aureus* DNA, plus one control pure aerosol sample) at 30°C for 16 hr using the REPLI-g whole genome amplification kit (Qiagen, Valencia, CA). The amplified material was inactivated at 65°C for 3 min and then purified using the QiaQuick PCR purification kit (Qiagen) to remove the primers and dNTPs. The entire amplified product was labeled with Cy3-random primer using the Klenow fragment and then hybridized to the array as described above.

For experiments on detection of virulence gene orthologs in related organisms, *E.coli* O157:H7 strain EDL933, *Staphylococcus saprophyticus* subsp. *saprophyticus* strain ATCC 15305, *Salmonella enterica* subspecies *enterica* serovar *Paratyphi A* strain ATCC 9150, and *Streptococcus pyogenes* strain MGAS5005 were purchased from ATCC. Samples were prepared from these organisms as described above for the other pure bacterial cultures.

### Statistical methods for data analysis

Data were analyzed using custom software based on the R programming environment and BioConductor packages. Each probe was randomly spotted in three to five replicates to control for positional effects on the array. Data from replicate probes were summarized by the median of the log_2_-transformed intensities. Each probe on an array was considered to have a positive signal if the median log_2_ intensity of its technical replicates was above a detection threshold calculated for that array. The detection threshold was determined by using random control probes to model background noise. For each array in the target probe specificity and limit of detection experiments, the detection threshold was set to the median log_2_ intensity of the random control probes, plus 4 times the standard deviation of the log_2_ intensities. For detection of virulence gene orthologs in organisms other than the sources of probe target sequences, a more stringent threshold defined as the 99^th^ percentile of the random control intensities was used.

### NCBI GEO submission

The data discussed in this publication have been deposited in NCBI's Gene Expression Omnibus (GEO, http://www.ncbi.nlm.nih.gov/geo/) and are accessible through GEO series accession number GSE11010.

## Results

### Target strain specificity

**Figure 4:**
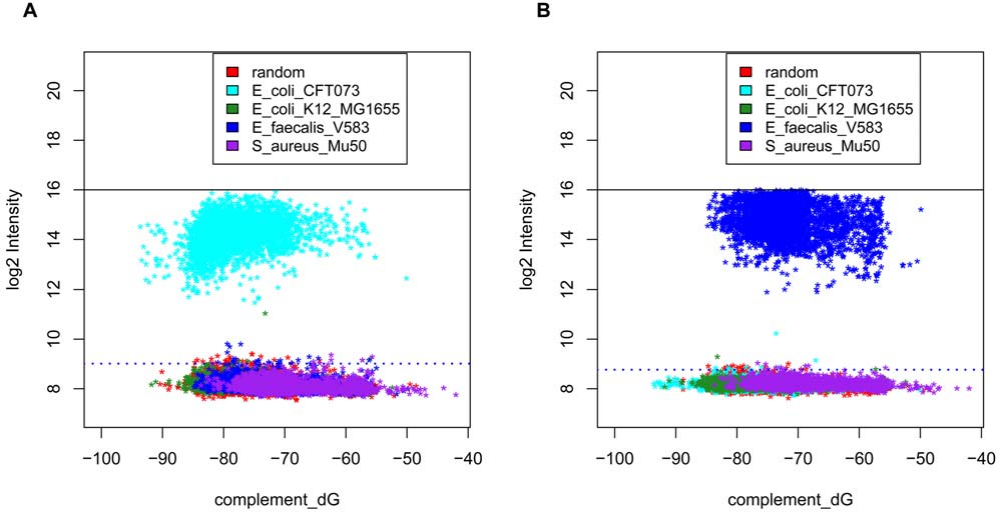
Hybridization of *E. coli* CFT073 and *E. faecalis* pure genomic DNA to NimbleGen virulence array. A. 4μg of Cy3-labeled *E. coli* CFT073 or B. *E. faecalis* DNA were hybridized to the array and the log_2_ intensity vs probe complement ΔG was plotted. Random control probes, *E. coli* CFT073, *E. coli* K12, *E. faecalis* and *S. aureus* strain –specific probes are shown in red, yellow, green, cyan and purple colors. The probes that are specific to virulence genes present in the target strain have much higher signal intensities than the random control probes and probes specific to the other three organisms. The detection threshold was set as median + 4 SD.

To assess the ability of the NimbleGen array to reliably identify a target organism of known genome sequence, we performed BLAST searches for all target probe sequences against the four genomes (sequences derived from GenBank), and selected subsets of probes that had a full length perfect match to one genome, and no perfect match longer than 16 nt to any of the other 3 genomes. We refer to these probes as strain-specific probes. We performed hybridizations with purified genomic DNA from either *E. coli* CFT073, or *E. faecalis* or *E. coli* K12. Figure 4 shows log_2_ intensities plotted against Δ*G_complement_* for the strain-specific probes for the *E. coli* CFT073 and *E. faecalis* hybridizations. The dotted blue line in each plot is the detection threshold for each array representing the median+4 SD of the negative controls (random sequence probes).

In each case, the probes that were specific to virulence genes present in the target strain had much higher signal intensities than the random control probes and probes specific to the other three organisms. The same pattern was observed in the hybridization with *E. coli* K12 (data not shown). The true positive rate of detection, measured by the fraction of probes specific to the hybridized strain with intensity over the threshold (median+4 SD) is 100%. The false positive rate, the fraction of intensities over the threshold for probes specific to a different strain, was only 0.29%.

### Detection of virulence genes from related organisms

In order to assess the ability of probes designed against gene family members from one organism to detect orthologs with non-identical sequences from other organisms, we performed two sets of experiments, one using the perfect match target probes, the other (to be discussed below) using the mismatch probes. In the first set of experiments, arrays were hybridized to DNA from four bacterial strains that do not have organism-specific probes on the array, as described in Methods. These four strains were chosen because they have fully sequenced genomes, are readily available from ATCC, and span a range of phylogenetic distances from the four target strains used to design probes on the array. One of these is a different strain of the same species (*E. coli*) as two of the target strains; one is a different species of the same genus (*Staphylococcus*) as a target strain; one belongs to a different genus (*Salmonella*) of the same family as *E.coli*; and one (*S. pyogenes*) belongs to a different family of the same order as *E.faecalis*. All four strains were found by our HMM analysis to possess orthologs for a variety of virulence gene families; through the results of this analysis, we were able to divide the 160 gene families with probes on the array into “present” and “absent” groups (i.e., families with or without orthologs) in a given strain.

In the analysis of arrays from this experiment, a gene family was considered “detected” if at least one probe specific for that family had median intensity above the detection threshold. A detailed listing of gene families, indicating the strains in which they are present and/or detected, is given in supporting information file Table S1. The results of this analysis are summarized in Table 3. For each array, sensitivity is calculated as the fraction of present families that were detected (true positives), while specificity is the fraction of true negatives among the absent families. The type I and type II error rates are calculated as the fractions of false positives and false negatives, respectively, among absent and present families.

**Table 3:** Virulence gene ortholog detection in four bacterial strains without genome-specific probes on the array.

	*E.coli O157:H7 EDL 933*	*S.enterica paratyphi ATCC 9150*	*S.saprophyticus ATCC 15305*	*S.pyogenes MGAS5005*
**Gene family counts:**				
Present in strain	159	143	50	32
Absent in strain	1	17	110	128
Detected	160	102	44	19
Not detected	0	60	118	143
True positives	159	99	34	9
False positives	1	1	8	8
True negatives	0	16	102	120
False negatives	0	44	16	23
**Detection and error rates:**				
Sensitivity	100.0%	69.2%	68.0%	28.1%
Specificity	0.0%	94.1%	92.7%	93.8%
Type I error	100.0%	5.9%	7.3%	6.3%
Type II error	0.0%	30.8%	32.0%	71.9%

In *E. coli* O157:H7 strain EDL933, 159 of the 160 gene families were represented by an ortholog. All of them were detected; in addition, an efflux pump protein family not present in this strain was also detected, yielding 100% sensitivity and 0% specificity rates.

In the other bacterial strains tested, the specificity averaged 93.5%, and sensitivity ranged from 69% to 28%, decreasing with the taxonomic distance of the test strain from the closest strain with perfect match probes on the array.

Our results from this small set of organisms suggest that probes designed according to our strategy against gene family members from one species can reliably detect orthologs in different species of the same genus, and even different genera of the same taxonomic family. Excepting the unusual case of *E. coli* O157:H7, the false positive rate was 7.3% or less in all hybridizations performed.

### Mismatch probe sensitivity

**Figure 5:**
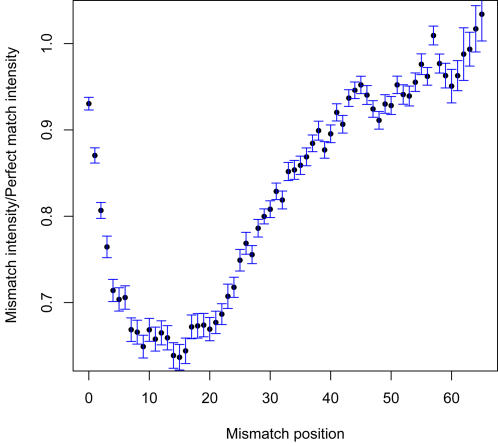
Effect of the position of mismatches on the hybridization of target and probe. The mean mismatch (MM) probe intensity vs perfect match (PM) probe intensity ratio, averaged over 60 PM probes and their corresponding MM probes, from 10 arrays is plotted vs the position of the MM.

To more comprehensively assess the factors influencing the balance between probe sensitivity and specificity, we analyzed data from two series of probes, containing single and multiple mismatches respectively. We first examined data from probes that perfectly matched the hybridized strain, except for a single mismatch (MM) base placed at a known position. We investigated the effect of the MM position on the probe intensity, relative to the intensity of the corresponding perfect match (PM) probe for the same hybridization. Figure 5 shows mean intensity ratios (MM/PM) with error bars corresponding to 95% confidence intervals, averaged over 60 PM probes and their cognate MM probes, from 10 arrays hybridized to a variety of samples. The MM positions are numbered in 5′ to 3′ order, and probe lengths range from 30 to 66.

As shown in this figure, single MM probe intensities varied with the position of the MM. On NimbleGen arrays, the 3′ end of the probe is attached by a 5-T linker to the glass surface of the array. We observed that mismatches located 7 to 20 nt from the 5′ end of the probe had the strongest negative impact on hybridization, while mismatches located on one of the 12 nucleotides closest to the linker had virtually no discernable effect. We also note that, even at the position of maximum effect, 15 nt from the 5′ end, single mismatches have relatively small impact, with only a 35% reduction of intensity relative to the corresponding PM probe. The reduction in intensity appears to be greater for shorter than for longer probes (data not shown).

In the single-MM experiments, MM probes were generated containing all three possible choices of MM base at each position. We found no consistent difference in intensity between probes using the complement of the PM base and probes generated by transition or non-complementary transversion of the PM base.

**Figure 6:**
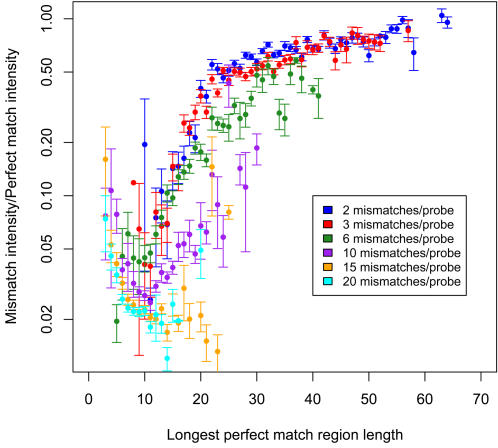
Effect of the length of perfect match (PM) sequence on the hybridization of target and probe. 2, 3, 6, 10, 15 and 20 mismatches were randomly created. Intensity ratios were averaged over 10 arrays for the 60 sets of PM and MM probes, and are plotted here on a log scale against the length of the longest PM region.

When probes contained multiple mismatches to the genome of the hybridized strain, we found that the reduction in intensity depended not only on the number of mismatched bases, but also on the length of the longest PM sequence between mismatches. Consequently, longer probes tend to be more tolerant of mismatches. The relationship between the number of MM bases, the longest PM region length, and the reduction in intensity relative to the cognate PM probe is shown in Figure 6. The graph shows the mean MM/PM intensity ratios averaged over 60 PM probes and the corresponding random MM probes. The random MM probes were generated from the PM probe sequences by selecting 2, 3, 6, 10, 15 or 20 random positions in the PM probe, and creating single mismatches at each position. Intensity ratios were averaged over 10 arrays for the 60 sets of PM and MM probes, and are plotted here on a log scale against the length of the longest PM region.

We observed that probes with two or three mismatches to the hybridized strain had at least half the intensity of the related PM probes, provided there was at least one PM region with length≥29 nt. This was nearly always the case for 60-mer probes. Probes with six mismatches had greater signal reduction, but still had 30% or more of the PM probe intensity when the mismatches were clustered toward one end of the probe, leaving a 29 nt or longer PM region.

Probes with 10 or more MM bases showed even greater signal reduction, and also more variability in reduction between probes. We conjecture that this variability is related to the position of the PM regions within the probe, with regions overlapping the 5′ half of the probe having a stronger positive effect on signal intensity. Additional experiments using a wider variety of MM configurations would be required to test this hypothesis adequately.

In general, all probes with PM regions≥29 nt had intensities above the detection threshold. Probes with shorter maximal PM regions were detected some of the time, but not consistently.

### Limit of detection of genomic DNA in an environmental sample background

Several experiments were performed to show the dynamic range and limit of detection of our array, along with its ability to identify specific organisms within a complex background, when combined with our protocol for sample preparation. We created six target microbial DNA samples using DNA isolated from an aerosol sample (24 hour filter collection from an urban environment) as complex background material. One target contained background DNA only; the others were spiked with fragmented *S. aureus* DNA in amounts ranging from 0.31 fg to 3.1 pg, amplified, labeled and hybridized to arrays, as described in Methods. For comparison, the complete 2.88 Mb *S. aureus* chromosome has a mass of about 2.95 fg.

**Figure 7:**
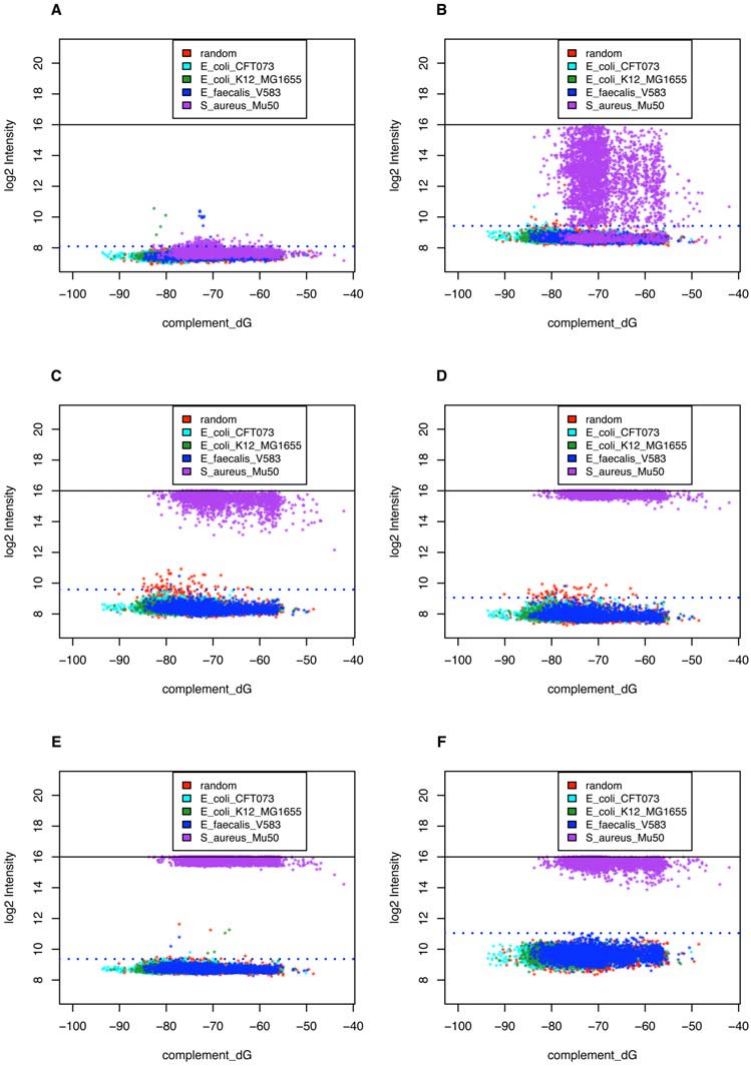
Detection of virulence genes from *S. aureus* spiked into BioWatch aerosol filter samples. 0.31 fg, 3.1 fg, 31 fg, 310 fg and 3.1 pg of *S. aureus* DNA were spiked into 10 ng of extracted BioWatch aerosol DNA samples. Aerosol sample alone or the mixed DNA samples were amplified, labeled and hybridized. Plots show log_2_ intensity of probes vs *ΔG _complement_*. Array hybridized with aerosol sample only is shown in Figure 7A and the arrays hybridized with 0.31 fg to 3.1 pg spiked in *S. aureus* are shown in Figures 7B-9F. 100% of the probes specific to virulence genes in *S. aureus* were detected in 3.1 fg and above. 36% of the probes were detected at 0.31 fg of *S. aureus*.

Figure 7 shows the intensity of strain-specific probes versus Δ*G_complement_* for arrays hybridized to each of the six samples. In the unspiked aerosol background DNA, we found only a few probes with signals barely above the detection threshold; therefore we expect that the signal seen in the spiked samples is mostly or entirely due to the added *S. aureus* DNA. With 0.31 fg of *S. aureus* DNA, we observe about 36% of *S. aureus*–specific probes with signals above the threshold. The detectable probes cover about 37% of the targeted virulence gene orthologs. This level of detection was reproducibly observed in multiple experiments. With 3.1 fg, we see that 100% of the *S. aureus* specific probes were above the detection threshold. With 31, 310 or 3100 fg, virtually all of the *S. aureus* specific probes were saturated, with intensities within a factor of two below the maximum possible intensity.

## Discussion

The emerging threat presented by novel pathogens, whether they arise naturally or are deliberately engineered, creates a need for detection systems that can warn public health authorities about a potential outbreak and help them select appropriate countermeasures. Ideally, such a system will be able to determine the virulence and antibiotic resistance mechanisms present in a sample of unidentified microorganisms, even when the sample includes organisms never previously encountered. As a step toward developing such a system, we have produced and tested a series of highly sensitive and specific functional gene arrays using the NimbleGen platform. These are the first functional gene arrays created that can quantify the presence or absence of hundreds of virulence gene families with a single assay. Our goals for this study were to develop methods for design of gene family-specific probes, to measure the sensitivity and specificity of these arrays, and to assess the validity of the FGA approach for detecting a broad spectrum of virulence and antibiotic resistance mechanisms in unknown as well as known microorganisms without culturing them.

The array described in this report includes probes for 1,245 target sequences, within genes belonging to 160 virulence and antibiotic resistance gene families, identified in *E. coli* K12, *E. coli* CFT073, *S. aureus* and/or *E. faecalis*. These sequences were selected using a collection of over 700 hidden Markov models, each of which was trained against sequences of a single virulence or antibiotic resistance gene family, identified by an extensive literature search. Using these models, more than 200,000 targets were identified in a database of bacterial, viral and other genome sequences; the sequences with probes on the current array are those identified in one of the four target strains. While this set of models targets a substantial fraction of the virulence and antibiotic resistance gene families known at present, future arrays in this series will be based on a comprehensive set of over 1,500 HMMs covering the majority of known virulence and A/R related genes. (McLoughlin, manuscript in preparation).

We used a novel approach to design groups of probes specific for virulence gene families. Prior to our study, there was no software available that could design minimal sets of family-specific probes for such a wide variety of sequences from unrelated organisms. Sequences within a gene family frequently are highly polymorphic at the nucleotide level, despite the functional conservation within the family. In order to minimize the total number of probes while covering as many families as possible across a phylogenetically diverse set of organisms, we developed rigorous algorithms to choose conserved probes that ensured detection of divergent sequences within a family.

We note that the optimal characteristics of probes for gene family detection differ greatly from those for applications such as gene expression, in which mismatch bases are not tolerated and ideal probes produce linear signals in response to target concentration. For gene family detection, probes are required to tolerate a certain number of mismatches, commensurate with the degree of polymorphism within a gene family, without cross-hybridizing to members of other families. Linearity of response is not a concern, since our goal is to measure presence rather than abundance; in fact, we prefer to have probes that saturate in response to small quantities of complementary DNA. For this purpose, it worked well to calculate predicted free energies of hybridization for candidate probes against their complements, along with free energies for homodimer formation and self-hybridization. By setting a minimum threshold for an empirically derived linear combination of these free energies, we were able to select probes that had the necessary degree of sensitivity and mismatch tolerance.

Because environmental samples may contain limited quantities of intact pathogen DNA, we expect that sample material will need to be amplified to generate the amount of target DNA required to produce a detectable signal on the array. Whole genome amplification has been used widely for bacterial genomes, producing high yields with low bias. In a study by Arriola *et al* for comparative genome hybridization microarrays of cancer samples, they have shown that the amplification bias using bacteriophage Phi29 polymerase is less than 0.5% when sufficient material is used [15]. Wu *et al* have reported that they were able to detect as little as 10 fg of microbial community DNA on their 50-mer functional gene array when combined with whole community genome amplification [16]. This amplification technique provides many advantages over specific amplification when the organism and mechanism to be identified are unknown, and appropriate culture techniques cannot be inferred. The amplified DNA can be directly labeled with fluorescent dyes and hybridized with high specificity to hundreds of thousands of probes on arrays.

In our own limit of detection study, we applied whole genome amplification to initial quantities of fragmented *S. aureus* DNA as low as 0.31 fg and hybridized the amplified DNA to our virulence mechanism array. We found we were able to detect 100% of the virulence and antibiotic resistance probes expected to be present in *S. aureus* in a sample amplified from 3.1 fg of starting DNA. Since the *S. aureus* strain used has a genome mass of 2.95 fg, this starting amount is equivalent to slightly more than one genome copy.

We used two approaches to assess the array's tolerance for mismatches between probe and target sequence. The first was to design more than 24,000 probes with mismatch (MM) nucleotides at known positions, and compare their performance to perfect match (PM) probes targeting the same bacterial sequences. We found that long oligomer probes with 50 to 66 nucleotides yielded greater than 90% detection rates, even with up to three mismatches from the target sequence. Probes with larger numbers of mismatches still gave high detection rates, provided there was a region of PM sequence with length at least 29 nt. A previous study by Kane *et al* showed that 50-mer probes, with 15-, 20- or 35-nt regions of PM to the hybridized genome sequence, had respective signal intensities approximately 1%, 4% or 50% of those obtained for perfect matches over the entire probe length [17]. He *et al* reported that 70-mer probes, when hybridized to DNA with PM sequence lengths of 20, 35 or 50 nt, yielded signal intensities 10%, 32% or 55% respectively of those obtained for full length perfect matches [18]. Our probes appear to have much better tolerance to stretches of mismatches. This could be due to the sensitivity constraints in our probe design algorithm, which favor probes with lower (more negative) hybridization free energies.

Higher MM tolerance was seen when the mismatches were placed nearer the 3′ end of the probe, which is anchored to the array surface. Conversely, the region of maximum sensitivity to mismatches is about 1/3 of the distance from the 5′ end to the 3′ end. This position dependence is not accounted for in current algorithms for prediction of hybridization free energies. Our probe design software for future generations of virulence gene detection arrays will factor in this dependence, placing more highly conserved regions of gene families in the areas of maximum impact along the length of the probe.

Our other approach to assess MM tolerance was to hybridize the array to genomic DNA from organisms with varying degrees of relatedness to the four target strains used for probe design. We found that a different *E. coli* strain from the two used for probe design still gave good results, with 100% of gene families detected by one or more probes. A *Salmonella* strain, which belongs to the same taxonomic family (*Enterobacteriaceae*) as *E. coli*, also had a large fraction of expected gene families (69%) with detection signals. Interestingly, the array performed almost equally well with a member of the *Staphylococcus* genus, with 68% of expected gene families being detected. These results may simply indicate that taxonomic categories are only a rough indication of phylogenetic relatedness. These preliminary results are encouraging, at any rate; they suggest that a future array with probes for each gene family sampled intelligently from the whole range of bacterial taxonomic families stands a reasonable chance of being able to detect orthologs from species that are not currently sequenced.

One of the limitations of functional gene arrays is that they cannot detect SNP-based or small indel-based mutations that affect virulence or resistance, because probes are selected to not be sensitive to small mutations. This is an unavoidable cost of designing an array to detect broad patterns of gene presence. Thus, an ideal platform for pathogen detection would pair the broad virulence mechanism array with a resequencing array for specific virulence genes, whose polymorphisms have well understood effects on virulence or drug resistance. In this scenario, the resequencing array would be used as a secondary analysis if the broad mechanism array indicated the presence of virulence genes known to have important sequence variations.

Finally, we emphasize the value of profiling multiple virulence related gene families in parallel, a unique advantage of microarray-based detection systems. Many virulence mechanisms require the coordinated actions of gene products from multiple gene families; therefore the presence in an organism of orthologs from most of the relevant families constitutes much stronger evidence for possession of a mechanism than the presence of one or two orthologs. We are developing analysis algorithms that will enable us to assign probabilities for the existence of particular virulence mechanisms in an unknown organism, using the unique discriminative power afforded by a multiple-family functional gene array.

The NimbleGen virulence gene array we developed shows great promise for detection of a broad range of virulence and antibiotic resistance genes. In addition to providing strain-level identification of known organisms, this technology will be valuable for functional characterization of unknown biothreat organisms. As a concrete example, we will use future versions of the array to identify or provide nearest-neighbor matches to organisms present in environmental samples collected by the BioWatch program (http://www.fas.org/sgp/crs/terror/RL32152.html), and to assess the pathogenic capabilities of unidentified organisms. Thus, our array can provide orthogonal confirmation for signature-based detection methods such as PCR. This array can also be used to differentiate virulent and avirulent strains by including antivirulence genes on the array [19]. Finally, the tools we have developed to design and analyze these arrays can be applied to create other kinds of functional gene arrays that will be valuable for the discovery of new pathogens, monitoring the metabolic capabilities of environmental and communal microbes, and performing functional forensic microbial analysis.

## Supporting Information

Table S1 Expected presence or absence of orthologs for target gene families and actual detection results for four organisms tested (E. coli O157:H7 EDL 933, S. enterica serovar paratyphi, S. saprophyticus. and S. pyogenes).
(0.09 MB XLS)
